# Parasitic helminth infections in humans modulate Trefoil Factor levels in a manner dependent on the species of parasite and age of the host

**DOI:** 10.1371/journal.pntd.0009550

**Published:** 2021-10-18

**Authors:** Babatunde Adewale, Jonathan R. Heintz, Christopher F. Pastore, Heather L. Rossi, Li-Yin Hung, Nurudeen Rahman, Jeff Bethony, David Diemert, James Ayorinde Babatunde, De’Broski R. Herbert

**Affiliations:** 1 Public Health Department, Nigerian Institute of Medical Research, Yaba, Lagos, Nigeria; 2 University of Pennsylvania, Perlman School of Medicine Biostatistics Analysis Center, Philadelphia, Pennsylvania, United States of America; 3 Department of Pathobiology, University of Pennsylvania School of Veterinary Medicine, Philadelphia, Pennsylvania, United States of Amerca; 4 Department of Medicine, Division of Experimental Medicine, University of California, San Francisco, San Francisco, California, United States of Amerca; 5 Department of Microbiology, Immunology & Tropical Medicine, George Washington University Medical Center, Washington, District of Columbia, United States of Amerca; 6 Department of Biochemistry & Nutrition, Nigerian Institute of Medical Research, Yaba, Lagos, Nigeria; NIH-National Institute for Research in Tuberculosis-ICER, INDIA

## Abstract

Helminth infections, including hookworms and Schistosomes, can cause severe disability and death. Infection management and control would benefit from identification of biomarkers for early detection and prognosis. While animal models suggest that Trefoil Factor Family proteins (TFF2 and TFF3) and interleukin-33 (IL-33) -driven type 2 immune responses are critical mediators of tissue repair and worm clearance in the context of hookworm infection, very little is known about how they are modulated in the context of human helminth infection. We measured TFF2, TFF3, and IL-33 levels in serum from patients in Brazil infected with Hookworm and/or Schistosomes, and compared them to endemic and non-endemic controls. TFF2 was specifically elevated by Hookworm infection in females, not Schistosoma or co-infection. This elevation was correlated with age, but not worm burden. TFF3 was elevated by Schistosoma infection and found to be generally higher in females. IL-33 was not significantly altered by infection. To determine if this might apply more broadly to other species or regions, we measured TFFs and cytokine levels (IFNγ, TNFα, IL-33, IL-13, IL-1β, IL-17A, IL-22, and IL-10) in both the serum and urine of Nigerian school children infected with *S*. *haematobium*. We found that serum levels of TFF2 and 3 were reduced by infection, likely in an age dependent manner. In the serum, only IL-10 and IL-13 were significantly increased, while in urine IFN-γ, TNF-α, IL-13, IL-1β, IL-22, and IL-10 were significantly increased in by infection. Taken together, these data support a role for TFF proteins in human helminth infection.

## Author summary

Billions of people are infected with parasitic helminths across the globe, especially in resource poor regions. These infections can result in severe developmental delay, disability, and death. Adequate management of helminth infection would benefit from the identification of host biomarkers in easily obtained samples (e.g. serum or urine) that correlate to infection state. Our goal was to determine if specific proteins involved in tissue repair and immune modulation are altered by infection with specific helminth species in Brazil (hookworm and *S*. *mansoni* species of blood fluke) or Nigeria (*S*. *haematobium* species of blood fluke). One of these proteins, Trefoil Factor 2 (TFF2), was elevated in the serum of hookworm infected women from Brazil, while another, TFF3 is higher in women than men, but also increased by *S*. *mansoni* blood fluke infection. In contrast, both TFFs were decreased in the serum of Nigerian children infected by *S*. *haematobium*, while many pro-inflammatory cytokines were increased in the urine, where the eggs emerge from host tissue.

## Introduction

Billions of people are infected with parasitic helminths including cestode, nematode, and trematode species that are collectively responsible for millions of disability associated life years (DALYs) annually across the globe [1]. While considerable advances have been made in drug development and mass drug administration efforts can reduce worm burdens in endemic areas, the high prevalence of re-infection in humans living in endemic countries [2–4] suggests worms have evolved multiple ways to subvert and/or avoid host immunity. For instance, infection of children with hookworms including *Necator americanus* and *Ancylostoma duodenale* infections causes anemia and failure to thrive [5–8]. In adults, hookworm infections have been linked with a generalized immunosuppression that may lead to reduced vaccine efficacy [9,10]. *N*. *americanus* experimental infections engage host immunoregulatory pathways driven by cytokines like interleukin 10 (IL-10) and transforming growth factor beta (TGF-β) [11], which could partially explain the ameliorative effect of hookworm infections in the context of autoinflammatory diseases, like celiac disease [12]. Whether such immunosuppression is due to host derived suppressive molecules and/or parasite derived factors released within the excretory secretory products remains unclear.

The immunoregulatory impact of parasitic helminths on their hosts is also a central feature of infections by blood flukes in the Schistosoma species. Human schistosomiasis, a neglected tropical disease that affects over 250 million people worldwide, results in severe morbidity, compromised childhood development, and an estimated 280,000 deaths annually [13]. Although praziquantel administration is an effective pharmacological treatment against adult Schistosomes [14], patients often become re-infected [15,16], which again points to the ability of Schistosomes to modulate the immunological landscape of their hosts during chronic parasitism. Indeed, humans infected with *S*. *haematobium* can mount prototypical Type 2 responses associated with reduced damage and parasite clearance [17], but also show reduced tendency toward allergic responses [18–20], implying that ongoing infection downmodulates the inflammatory status of the host. Further, the generation of long-lasting immunotherapies to combat Schistosoma infections is greatly limited by our poor understanding of how helminth-induced inflammation is regulated. A greater understanding of how different worm species module their hosts is certainly needed in order to address three unresolved issues: 1) does parasite burden correlate with or predict the degree of immunomodulation, 2) do humans living in distinct endemic areas mount similar responses, 3) how does tissue injury caused by parasitic infection elicit host immune and tissue repair responses?

Amongst all of the known tissue repair mechanisms operating at the mucosal interface, Trefoil factor family proteins (TFF1, TFF2, and TFF3) remain one of the most poorly understood. TFF proteins are small secreted glycoproteins produced by goblet cells under both homeostatic and injury-induced conditions [21–23]. Trefoils are named after their evolutionarily conserved cloverleaf shaped “P” or trefoil-domain, which imparts functional resistance to proteolysis [24]. TFF2 and TFF3 are the predominant TFFs produced in the colon of humans and most mammals, but only bear ~20% amino acid conservation [25]. TFFs 1–3 are diagnostic for GI tissue injury responses in human mucosa, e.g., TFF expression marks the ulcer-associated cell lineage (UACL), which defines cells located at the regenerative border of GI ulcers [26,27]. Although it is known that prophylactic administration of rTFF2 and rTFF3 into the GI lumen of rodents with injury induced inflammation leads to suppression of disease [28–30], the lack of protective efficacy for TFF3 enema administration in a clinical trial of humans with Ulcerative Colitis [31] has led to controversy regarding the necessary context and utility for using TFFs in the regulation of GI inflammatory disease. Our work has implicated TFF’s as important immunoregulatory molecules in the context of GI parasite infection [32–35]. Importantly we found that TFF2 can suppress proinflammatory cytokine production in the context of *T*. *gondii* infection [34] and the genetic deficiency in TFF3 led to increased release of Type 1 inflammatory cytokine INFγ [32].

The overarching goal of this study was to address the idea that the chronic injurious nature of hookworm or Schistosoma infection in different parts of the world would associate with changes in TFF2 or 3. We also sought to determine if parasite burden might correlate with the levels of TFFs. Here, we find that hookworm infection in a Brazilian cohort preferentially elevated TFF2 levels in females, even when compared to co-infection with Schistosomes or Schistosoma alone. In contrast to our expectation, we found that older age, rather than egg burden had a stronger positive correlation with TFF2 levels. Serum TFF3 was generally elevated in Brazilian females and significantly increased by Schistosoma infection only. Interestingly, exposure of human PBMC from normal subjects not living in endemic areas shows that TFF2, but not TFF3 had suppressive effects on the ability of these cells to undergo PHA-induced proinflammatory cytokine production (TNFα and IFN-γ). Children in Nigeria with *S*. *haematobium* infection exhibited lower levels of serum TFF2 and TFF3 than uninfected children and higher levels of IL-10 and IL-13, which corresponded with higher levels of cytokines in the urine than those not infected, including the type 2 cytokine IL-13, the proinflammatory cytokines TNFα and IL-1β, and the regulatory cytokine IL-10. There was also a significant association of infection with the presence of TNF-α, INF-γ, and IL-22 in the urine. Taken together, this work indicates that TFF levels are modulated in body fluids of infected individuals and may have a role in promoting immunoregulation that occurs in such individuals.

## Methods

### Ethics statement

These studies were approved by the George Washington University (under approval numbers: IRB# 100310, IRB# 190988) and the Nigerian Institute of Medical Research Institutional Review Boards (IRB) (under approval number: No. IRB/18/042). All adult human subjects provided formal written informed consent and in the case of pediatric subjects, formal written consent of the parent or guardian was obtained, as well as assent of the minor subject.

### IRB approval and recruitment of samples from human subjects

The George Washington University IRB approved the project (IRB# 100310), which was effective August 22, 2011. Samples for this study were recruited from this ongoing study from 2013–2014. The study was conducted in an area endemic for hookworm in Americaninhas, Novo Oriente, Northeastern Minas Gerais, Brazil. All communication about the study, including the written consent form was conducted in Portuguese. The Brazilian Ministry of Health (MOH) and local health officials from each village were in charge of the on-site medical supervision. These officials routinely supply and administer anti-helminthics, and are proficient and skilled at drawing blood in a rural setting, due to continuous surveillance studies run by the MOH. Prior to obtaining written consent, subjects were informed of the study during a village meeting, when members of the local departmental health institutes provided an explanation about the aim, execution plan, and methodologies of the study. At this meeting the villagers were able to ask questions and offer their opinions, and efforts were made to ensure that the village residents understood, including their right to refuse participation in the study. After this meeting, written consent was obtained from all adult subjects, from the parents or guardians of minor subjects, and written assent was obtained from the minor subjects. Exclusion criteria included full time school or work attendance outside of the endemic area, a positive pregnancy test, or hemoglobin < 80g/L. Following positive diagnosis of infection, individuals were informed and offered treatment.

The George Washington University IRB also approved an additional protocol (IRB# 190988) to recruit healthy adult subjects (18–50 years old) to provide non-endemic control serum samples via word of mouth and advertisement (e.g. newspaper, online, Research Match). These subjects were also informed of the purpose of the study via in person meeting with a member of the study team in a private room, who also answered any questions the subject might have and stress that participation is voluntary. Subjects also receive a written description of the study and provided their signature on an informed consent form. It was made clear that they could withdraw their sample at any time, and the written document provided a means to contact the research team if they chose to revoke consent. Blood collection was conducted by a trained individual and the volume of collection did not exceed 50 mL.

The Nigerian Institute of Medical Research IRB approved the project (No. IRB/18/042), which was effective April 4, 2018 –March 10, 2019, when patient information and samples were recruited. Social approval for the study was obtained from the Medical Officer in-charge of Health in the Local Government as well as the approval of the Education Secretary of the Borgu Local Government Education Authority. Details of the procedure were explained to all participants during the social mobilization stage. The written informed consent of parents was obtained with the assent of the children to participate in the exercise. Participation was voluntary and the assent of each child was obtained before sample collection. The study was conducted in accordance with the tenets of Helsinki Declaration of 1964 as amended in 2013 and guidelines of Good Clinical Practice. Subjects were informed of infection and offered treatment.

### Patient demographics and samples

#### Brazilian cohort

Patients were recruited from hookworm (*N*. *americanus)* endemic areas for this study, consented to provide serum and fecal samples (to diagnose infection and assess egg burden). TFF2, TFF3, and IL-33 were measured from serum samples by ELISA. The first experiment assessed co-infection state, which is common in the region, and included patients infected with either Schistosoma (n = 11, 6 female), Hookworm (n = 20, 9 female), or both (n = 16, 8 female), and were compared to both uninfected endemic (n = 25, 13 female) and non-endemic (n = 20, 10 female) controls. This cohort ranged 5–67 years old (27 mean ± 18 SD years). We also collected from a larger cohort of hookworm-infected individuals that included 69 females (3–72 years old, 29 mean ± 21 SD years) and 97 males (4–70 years old, 27 mean ± 20 SD years), although sex identification was not tied to the other variables, so this could not be assessed in this dataset.

#### Nigerian cohort

Children aged 6–17 years undergoing routine health screening through their school provided blood, urine and feces, and were divided into infected or uninfected groups based on the presence of eggs from the blook fluke *S*. *haematobium* in urine samples. Fecal samples were used to determine if any control individuals (no *S*. *haematobium* eggs in urine) also had hookworm infection and these were excluded from analysis. There were no *S*. *haematobium* infected children co-infected with hookworm. Trefoil factors and cytokines (IL-33, IL-1b, IL-13, IL-17A, IL-22, IL-10, IFNγ and TNFα) were measured from serum and urine samples by ELISA. Dipstick uranalysis was performed by pipetting drops of urine onto Multisitx 10SG reagent strips from Siemens (Tarrytown, NY), to assess for signs of damage to the urinary tract such as elevated protein and presence of blood in the urine, and other features (specific gravity, pH, ketones, glucose, nitrite, urobilinogen, bilirubin and leukocyte content). Specific gravity (1.012 ± 0.001 uninfected vs 1.015 ± 0.002 infected; mean, SEM) and pH (6.6 ± 0.1 uninfected vs 6.6± 0.2 infected; mean, SEM) did not significantly differ between infection groups. Ketones were negative or 15 (n = 1/infection group). Glucose readings were negative for all samples. 3/48 uninfected and 1/17 infected samples were positive for nitrite. Urobilinogen values for all samples were 0.2. Bilirubin was small or negative for all samples. Leukocytes were moderate in 3/17 infected samples and registered no greater than small (n = 1) or trace (n = 3) in uninfected samples (n = 48 total).

In total, 78 (30 female) children, aged 6–14 years, provided samples. Preliminary laboratory analysis done at the Nigerian Institute of Medical Research was included prior to shipment to the University of Pennsylvania, where ELISA measurements were performed. After shipment, some samples lacked sufficient volume for testing. Of the serum samples tested, 51 came from children without *S*. *haematobium* eggs in their urine (23 were female, 17 had hookworm eggs in feces), 16 came from infected children with *S*. *haematobium* eggs in their urine (2 were female). Of the urine samples tested, 48 came from children without *S*. *haematobium* eggs in their urine (21 were female, 14 had hookworm eggs in feces), 17 came from infected children with *S*. *haematobium* eggs in their urine (23 were female). Only hookworm uninfected controls (n = 34) were compared to *S*. *haematobium* infected samples (n = 16–17).

### Enzyme linked immuno-sorbent assays (ELISAs)

Commercially available ELISAs were used to measure levels of human TFF2, TFF3, IL-33, IL-1β, IL-13, IL-17A, IL-22, IL-10, IFNγ and TNFα in human serum and urine samples, and for IFNγ and TNFα, media from control or stimulated human PMBCs, according to the manufacturer’s instructions. Human TFF2, TFF3, and IL-33 ELISA kits were from R&D Systems (Minneapolis, MN). Human IL-1β, IL-10, IL-22, IFN-γ, and TNF-⍺ ELISA kits were from Biolegend (San Diego, CA). Human IL-13 and IL-17A ELISA kits were from eBioscience (San Diego, CA). Because the Brazilian cohort required multiple plates to process the samples, standard curves and positive and negative plate to plate quality controls were run on each plate to ensure consistency across plates. All standard curves produced R squared values ranging from 0.978–0.993, and the quality controls were less than 2 standard deviations from the average across plates, indicating consistent performance across plates.

### Culture and treatment of human peripheral blood mononuclear cells

Human Peripheral Blood Mononuclear Cells (PBMCs) were obtained from four anonymous donors and generously provided by Dr. Douglas Nixon. Each donor’s cells were divided across 9 wells. Cells were plated 2.5 x 10^5^ per well in RPMI buffer (Gibco, Amarillo, TX) containing 5–10% human non-autologous plasma (Gemini Bio, West Sacramento, CA) and maintained in 37°C incubation with 5% CO_2_. Two wells per donor were pre-treated with human recombinant TFF2 or 3 overnight (25 ng/μL, US Biologicals, Salem, MA), before being stimulated with phytohemaglutinin (PHA, 50μg, Thermofisher, Waltham, MA), for 24 hours. Unstimulated controls were run simultaneously (one well per donor), both with or without exogenous TFFs, to confirm a lack of direct effect on cytokine release. For media, TFF2 and TFF3 only treatments, one well each was used per donor, and for PMA-stimulated treatments, two wells (technical replicates) each were used per donor. Supernatants were collected and IFNγ and TNFα were measured by ELISA at the end of stimulation. Each treatment point is the average of two duplicate wells. For culture data, which does not conform to normality, non-parametric comparison of three groups was performed using the Kruskal-Wallis test, with post-hoc Dunn’s multiple comparisons test.

### Statistical analysis

Statistical Analysis was performed using R and SAS. Multiple statistical models, methods, and/or transformations were employed due to inherent differences in the data for each analyte in both countries. The coding of age varied between the Brazil and Nigerian data: Age was continuous for the former and categorical (three bins 6–8, 9–11, or 12–14 years) for the latter.

#### Brazilian cohort

This cohort consisted of two datasets. One dataset included analytes (TFF2, TFF3, and IL-33) measured in serum from individuals with single or co-infections (referred to as “cohort”), with age (continuous) and sex included as fixed effects. The other included TFF2 measured in serum from individuals with hookworm infection only, with fecal egg count and age included as variables. For the first dataset, Analysis of Co-Variance (ANCOVA) was used to model TFF2, TFF3 and IL-33 with all 2- and 3-way interactions. More parsimonious models were tested by iteratively removing and/or adding fixed effects and interactions based on complexity and significance. If age and all respective interactions were nonsignificant, 1- and 2- way Analysis of Variance (ANOVA) models were constructed for the remaining covariates and interactions. All TFF3 and IL-33 models required transformations on the analyte to correct normality of the residuals: Using the Box Cox Procedure, TFF3 models were best corrected with log transformations while IL-33 models were best corrected with a reciprocal square root function. Normality was subsequently retested with the Shapiro-Wilk Test and was always nonsignificant. For the second dataset testing TFF2 and fecal egg counts, a regression was created with the covariate of age. Since fecal egg count was not significant, a regression was created between TFF2 and age, with TFF2 transformed with a reciprocal square root function to correct for normality of the residuals.

#### Nigerian cohort

Unfortunately, due to some loss of sample volume during shipping we were unable to measure all analytes for both urine and serum for every subject. Missing data, combined with the more limited sample size in this cohort meant that associations between each analyte and the covariates *S*. *haematobium* infection, sex, and age (categorical) could not be tested concurrently with 2- and 3-way ANOVAs, nor could interactions be tested formally. Instead, for each analyte, three 1-way ANOVAs were created testing each covariate individually; interactions were graphically assessed informally. Multiple imputation was considered to simulate missing values but was ultimately rejected for two reasons: a) most missing values were response variables (i.e., analytes) and b) there seemed to be dependence of missingness, meaning if one analyte was missing for an observation, all analytes were missing, consistent with our shipping difficulties [36]. That being said, the observations lost appeared to occur at random. Efforts were made to employ similar strategies for in serum and urine samples of the same marker, so they could be compared. For TFF2 in serum and urine and TFF3 in serum, the conditions for ANOVA were met, so we conducted several a one-way ANOVAs to test association between the analyte and each covariate of interest, making a Tukey’s adjustment for pairwise comparison of age categories. TFF3 Urine samples appeared relatively dichotomous (about 2/3 were less than 130pg/ml while the rest were above 2880pg/ml), and thus Chi-Squared Tests were conducted, testing for association between individual covariates and low/high TFF3 levels. For IL-13 and IL-1β in urine and Il-10 and IL-17A in serum, normality of the residuals was violated to an uncorrectable extent, so a Kruskal Wallis Test was conducted, followed by Wilcoxon Two-Sample Tests for pairwise comparisons of age groups. Several analytes had zero-inflated data, which is biologically sound in uninfected children, but statistically means that basic transformations and standard parametric tests are not feasible. A two-step model was created for IL-13 and TNF-α serum, IL-10 urine. First, a logistic model tested for an association between the binary presence of the marker with each covariate. Second, a Kruskal Wallis Test (since normality was violated in each case) was conducted for non-zero markers. Other markers (IL-33 serum and urine, TNF-α urine, INF-γ serum and urine, IL-17A urine, and IL-22 urine) had more zero-inflated data such that the conditions of logistic regression were violated; therefore, an association between the presence of the analyte and each covariate was tested with Pearson’s Chi Squared Test, Fisher’s Exact Test, means row test, or a test of non-zero correlation depending on the covariate of interest and requisite conditions. IL-1 β and IL-22 in serum could not be tested for any association due to the vast majority of analyte data equaling zero.

Because we were testing analytes in both urine and serum, we wanted to determine if there was any relationship between each analyte in these two sampling compartments where possible. For TFF2, a simple regression was conducted, while a two-step model was created for IL-13 (zero-inflated serum levels) and IL-10 (zero inflated urine levels), with the first step testing for association with logistic regression between the binary presence of the zero inflated marker and its respective counterpart and the second step testing association of the non-zero data. A one-way ANOVA was conducted for TFF3 and IL-17A: TFF3 appeared dichotomous while IL-17A urine was categorized as zero or nonzero. As mentioned, we were not able to do this for IL-1 β and IL-22, due to zero inflated data. For all others, an association was tested between the binary presence of both serum and urine markers.

## Results

### Helminth infections differentially modulate TFFs and hookworm single infection increases TFF2 age-dependently in the Brazilian cohort

**Fig 1:**
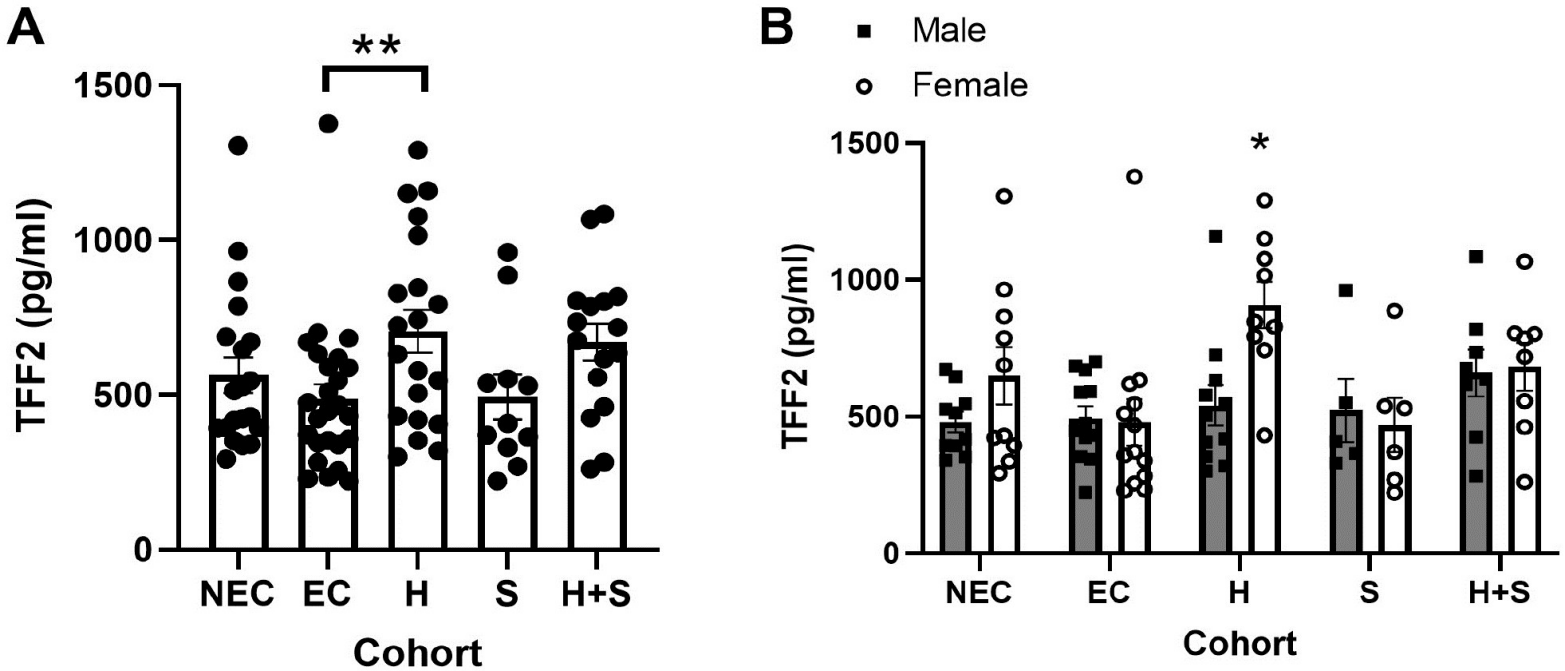
TFF2 levels are elevated by Hookworm infection in females from Brazil. (A) Levels of TFF2 in cohorts with sexes pooled or (B) with sexes shown separately in serum samples from Brazilian patients with either Schistosoma (S, n = 11, 6 female), Hookworm (H, n = 20, 9 female), or both (H+S, n = 16, 8 female) versus uninfected controls (endemic, EC, n = 25, 13 female and non-endemic, NEC, n = 20, 10 female). * p<0.05 for female H vs male EC, male NEC and female EC, ** p<0.01, post-hoc Tukey’s test.

**Fig 2:**
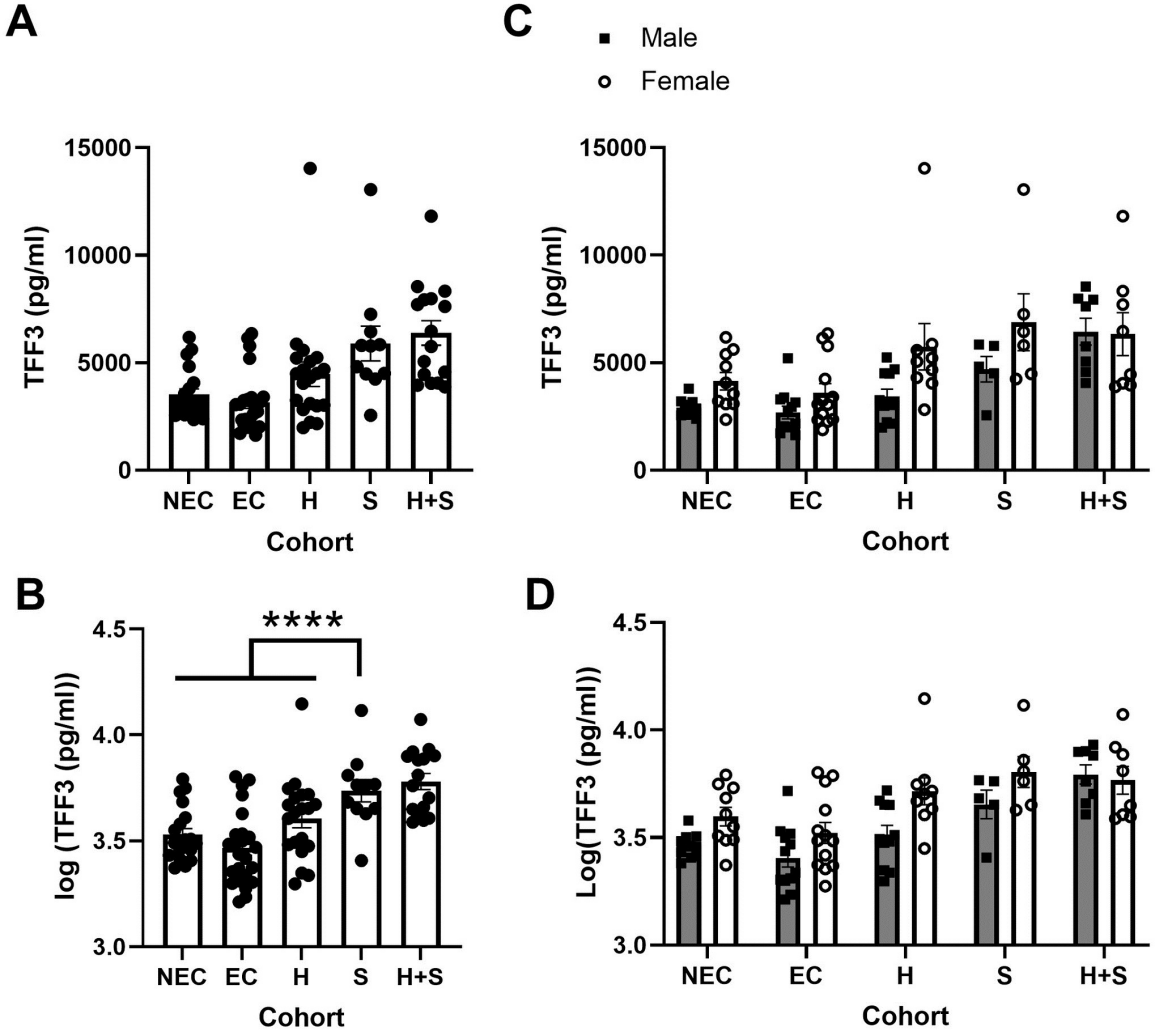
TFF3 serum levels are higher in Schistosoma infection than Hookworm or control in individuals from Brazil. (A,B) Levels of TFF3 and log transformed data in cohorts with sexes pooled or (C,D) with sexes shown separately in serum samples from Brazilian patients with either Schistosoma (S, n = 11, 6 female), Hookworm (H, n = 20, 9 female), or both (H+S, n = 16, 8 female) versus uninfected controls (endemic, EC, n = 25, 13 female and non-endemic, NEC, n = 20, 10 female). **** p<0.0001 for three indicated groups vs Schistosoma infection.

We have previously shown critical roles for TFF2, TFF3 and the alarmin cytokine IL-33 in protective immune responses in a murine model of hookworm infection [32,33,37], which needs to be confirmed in human infection. We first addressed this in patient samples from Brazil. Co-infection of helminth species is quite common in Brazil, particularly between hookworm (*N*. *americanus*) and *Schistosoma mansoni* [38], but the effects of these infections on TFFs or IL-33, either alone or together is unknown. Therefore, we sought to determine what effect co-infection of hookworm and Schistosoma, or either infection alone might have on TFF2, TFF3, and IL-33 levels in serum as compared to both endemic and non-endemic controls, accounting for age and sex as covariates. Age was not a significant factor for any of the analytes in this dataset. However, there was a significant effect of infection on both TFF2 (Fig 1, F = 3.23 p = 0.015) and TFF3 after transformation (Fig 2, F = 12.04, p <0.0001), but not on IL-33 levels after transformation (S1 Fig, F = 1.88 p = 0.1031).

For TFF2 controlled for sex, the analysis indicated that Hookworm infected individuals have significantly higher serum TFF2 levels than endemic controls (Fig 1A, Table 1). In the 2-way ANOVA, sex was not significant (F = 0.93 p = 0.3382), but there was a significant sex by cohort interaction (Fig 1B, F = 2.70 p = 0.0342), indicating that the effect of infection on TFF2 levels is dependent on sex. There is a significant difference between men and women in the hookworm cohort, and the difference between men and women in the Schistosoma cohort is trending toward significance (Table 2). Further, at least 1 cohort is significantly different within the female group (Table 3). Using pairwise tests of sexes and cohort, females infected with Hookworm had significantly elevated TFF2 levels when compared to female endemic controls (Tukey adjusted p-value = 0.0205), male endemic controls (Tukey adjusted p-value = 0.0366), and male non-endemic controls (Tukey adjusted p-value 0.0393) (Fig 1B). Overall, this indicates that TFF2 is elevated in Hookworm infected females, but not in Schistosoma or co-infection.

**Table 1:** Pairwise Tukey’s Post-hoc of TFF2 levels, sexes pooled.

Cohort 1	Cohort 2	Diff. of Means	P-value	95% Confidence Intervals
NEC	EC	77.833	0.8717	(-146.592, 302.259)
NEC	H+S	-52.016	0.964	(-269.856, 165.824)
NEC	H	-184.704	0.1505	(-406.742, 37.334)
NEC	S	-68.285	0.9305	(-305.363, 168.792)
EC	H+S	-129.850	0.4028	(-334.539, 74.840)
EC	H	-262.537	0.0063**	(-471.689, -53.386)
EC	S	-146.119	0.3787	(-371.173, 78.935)
H+S	H	-132.688	0.367	(-334.756, 69.381)
H+S	S	-16.269	0.9996	(-234.756, 202.218)
H	S	116.419	0.5972	(-106.255, 339.091)

Schistosoma (S, n = 11, 6 female), Hookworm (H, n = 20, 9 female), or Hookworm+Schstiosoma (S+H, n = 16, 8 female) versus uninfected controls (endemic, EC, n = 25, 13 female and non-endemic, NEC, n = 20, 10 female)

**Table 2:** Effect of sex within each cohort on TFF2 levels.

Cohort	DF	Sum of Squares	Mean Square	F Value	Pr > F
Nonendemic Control	1	142745	142745	1.96	0.1641
Endemic Control	1	1416.74881	1416.74881	0.02	0.8893
Hookworm/Schistosoma	1	24570	24570	0.34	0.5623
Hookworm	1	445948	445948	6.13	0.0148*
Schistosoma	1	269887	269887	3.71	0.0566

**Table 3:** Effect of cohort within each sex on TFF2 levels.

Sex	DF	Sum of Squares	Mean Square	F Value	Pr > F
Female	4	1042715	260679	3.58	0.0087**
Male	4	526891	131723	1.81	0.1317

For TFF3 controlled for sex, the analysis indicated that Schistosoma infected individuals had significantly higher levels than Hookworm infected or either control group (Fig 2A and 2B Table 4). In the 2-way ANOVA, there was a significant effect of sex overall (F = 29, p <0.0001), but no significant sex by cohort interaction, indicating that females have significantly higher TFF3 in general (Fig 2C and 2D). Overall, TFF2 elevation is specifically associated with Hookworm infection, especially in females, while TFF3 elevation is associated with Schistosoma infection and females have higher levels in general in a mostly adult population.

**Table 4:** Pairwise Tukey’s Post-hoc of log TFF3 levels in cohort groups, sexes pooled.

Group 1	Group 2	Diff. of Means	P-Value	95% Confidence Interval	Ratio group1/group2
NEC	EC	0.158	0.3381	(-0.076, 0.393)	1.172
NEC	H+S	-0.176	0.5017	(-0.481, 0.129)	0.839
NEC	H	-0.036	0.9892	(-0.248, 0.175)	0.964
NEC	S	-0.486	< .0001****	(-0.714, -0.259)	0.615
EC	H+S	-0.334	0.0607	(-0.678, 0.009)	0.716
EC	H	-0.195	0.2881	(-0.469, 0.079)	0.823
EC	S	-0.645	< .0001****	(-0.928, -0.361)	0.525
H+S	H	0.140	0.7799	(-0.197, 0.476)	1.150
H+S	S	-0.310	0.0955	(-0.653, 0.032)	0.733
H	S	-0.450	< .0001****	(-0.720, -0.180)	0.638

Schistosoma (S, n = 11, 6 female), Hookworm (H, n = 20, 9 female), or Hookworm+Schstiosoma (H+S, n = 16, 8 female) versus uninfected controls (endemic, EC, n = 25, 13 female and non-endemic, NEC, n = 20, 10 female)

**Fig 3:**
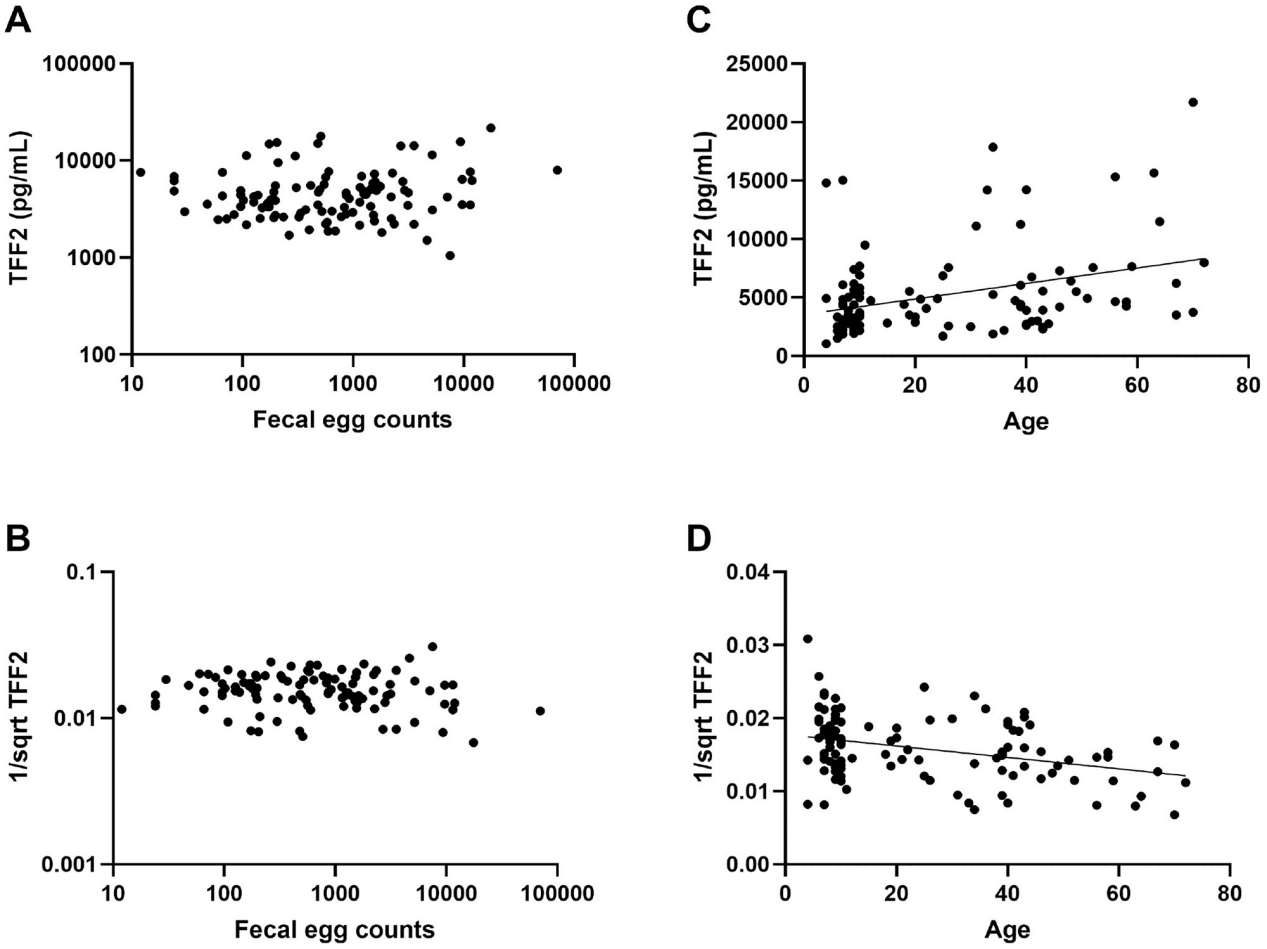
Relationship between TFF2 levels and serum and either Hookworm egg burden or age in a larger cohort of Hookworm-infected Brazilians. (A, B) Fecal egg counts plotted against either (A) TFF2 levels (pg/mL) in serum or (B) the transformed data required for statistical analysis. (C, D) Age plotted against either (C) TFF2 levels (pg/mL) in serum or (D) the transformed data.

Because Hookworm infection was associated with higher levels of TFF2, but individuals may have varying degrees of parasite burden, we collected a second larger dataset and used a regression between serum TFF2 and fecal egg counts with the covariate of age (Fig 3). Using an omnibus F-Test, fecal egg counts were not significantly correlated with transformed (reciprocal square-root) TFF2 levels in serum (p = 0.7655). However, in this large dataset, age was significantly associated with transformed TFF2 levels (p = 0.0002). This indicates that age, rather than worm burden, is more positively correlated with TFF2 levels in hookworm-infected individuals.

### TFF2, but not TFF3, suppresses pro-inflammatory cytokine release

Because TFFs are altered by helminth infection and known to modulate immune responses, we sought to determine what effect TFFs might have on proinflammatory responses. We cultured human PBMCs and stimulated them with phytohemaglutinin (PHA), both alone and in the presence of recombinant human TFF2 or TFF3. PHA mimics a highly inflammatory state and produces a significant increase in both interferon gamma (IFN-y) and tumor necrosis factor alpha (TNF-a) production vs media alone, or with TFFs alone (S2 Fig). This response was significantly repressed by co-treatment with rh-TFF2, but not rh-TFF3 (S2 Fig), indicating that elevated TFF2 could contribute to suppressed inflammatory responses in the context of hookworm infection.

### TFF serum levels are decreased with *S*. *haematobium* infection in Nigerian school children

Helminths impact a large proportion of the globe and a wide range of age groups. Nigeria is another region where different helminth infections are common, including the blood fluke *Schistosoma haematobium*, which is more prevalent among Nigerian school children than *S*. *mansoni* [39]. Given the differences we observed in the Brazilian cohort between hookworm and Schistosoma infections, we also sought to characterize TFF2, TFF3, and IL-33 levels, along with additional cytokines (IFNγ, TNFα, IL-13, IL-1β, IL-17A, IL-22, and IL-10) in a pediatric cohort from Nigeria including children infected with *S*. *haematobium*. This species of blood fluke infects and causes damage to the urinary tract [40], therefore we reasoned that serum and urine may provide different information and that it may be useful to determine if analytes correlate in the two compartments. We tested for associations between serum and urine levels of each analyte, but observed only three significant associations, all of which required categorization of at least one sample type: The presence of IL-13 in serum was associated with the IL-13 in urine (logistic regression p-value = 0.0203) while nonzero IL-13 in serum was associated with IL-13 in urine (linear regression p-value = 0.0107), the presence or absence of IL-17A in urine was associated with IL-17A in serum (Kruskal Wallis Test p-value = 0.0228), and the presence or absence of IL-33 in serum was associated with its presence or absence in urine (Fisher’s Exact Test p-value = 0.0236). Thus, we saw no evidence to suggest a direct correlation between continuous, quantitative levels of urine and serum samples of any analyte, and since analytes’ serum and urine levels do not appear related to each other, one cannot substitute for the other.

Because these samples come from children of mixed sex and age, we analyzed our findings to determine if sex or age influenced analyte levels. We did not observe significant effects of sex on any of the analytes measured in serum or urine, with the exception of IL-33 in urine (Fisher’s Exact test, p = 0.0254) and IFN-γ in serum (Pearson Chi-Square Tests of Independence, p = 0.0123), and in neither of these cases was infection associated with significantly altered levels. However, for age category, we did observe some significant associations for TFF2 and 3 in serum, and for some of the cytokines (e.g. IL-13, 1L-1β, and IL-10). However, given that the age range is limited and the categories are only 3-year bins (6–8, 9–11, and 12–14 years), any effect of age on analyte levels should be interpreted with caution (Table 5).

**Table 5:** Analytes and sample type with significant infection effect where age-effects were also observed in Nigerian children.

Analyte	Sample	Test	P-Value	Age-Group Pairwise	Difference of Mean^F^ or Medians^K-W^	P-Value
TFF2	Serum	F	0.0479	6–8 vs 9–11	138.07	0.0391
TFF3	Serum	F	0.0028	6–8 vs 12–14	150.27	0.0029
				9–11 vs 12–14	155.39	0.0254
IL-10	Serum	Kruskal-Wallis	0.0129	6–8 vs 12–14	-59.75	0.0021
IL-10*	Urine	Kruskal-Wallis	0.0103	6–8 vs 12–14	-10.87	0.0026
IL-1β	Urine	Kruskal-Wallis	0.0082	6–8 vs 12–14	-33.32	0.0139
				9–11 vs 12–14	-38.97	0.0078
IL-13	Urine	Kruskal-Wallis	0.0121	9–11 vs 12–14	-10.87	0.0028

*For non-zero data only.

**Fig 4:**
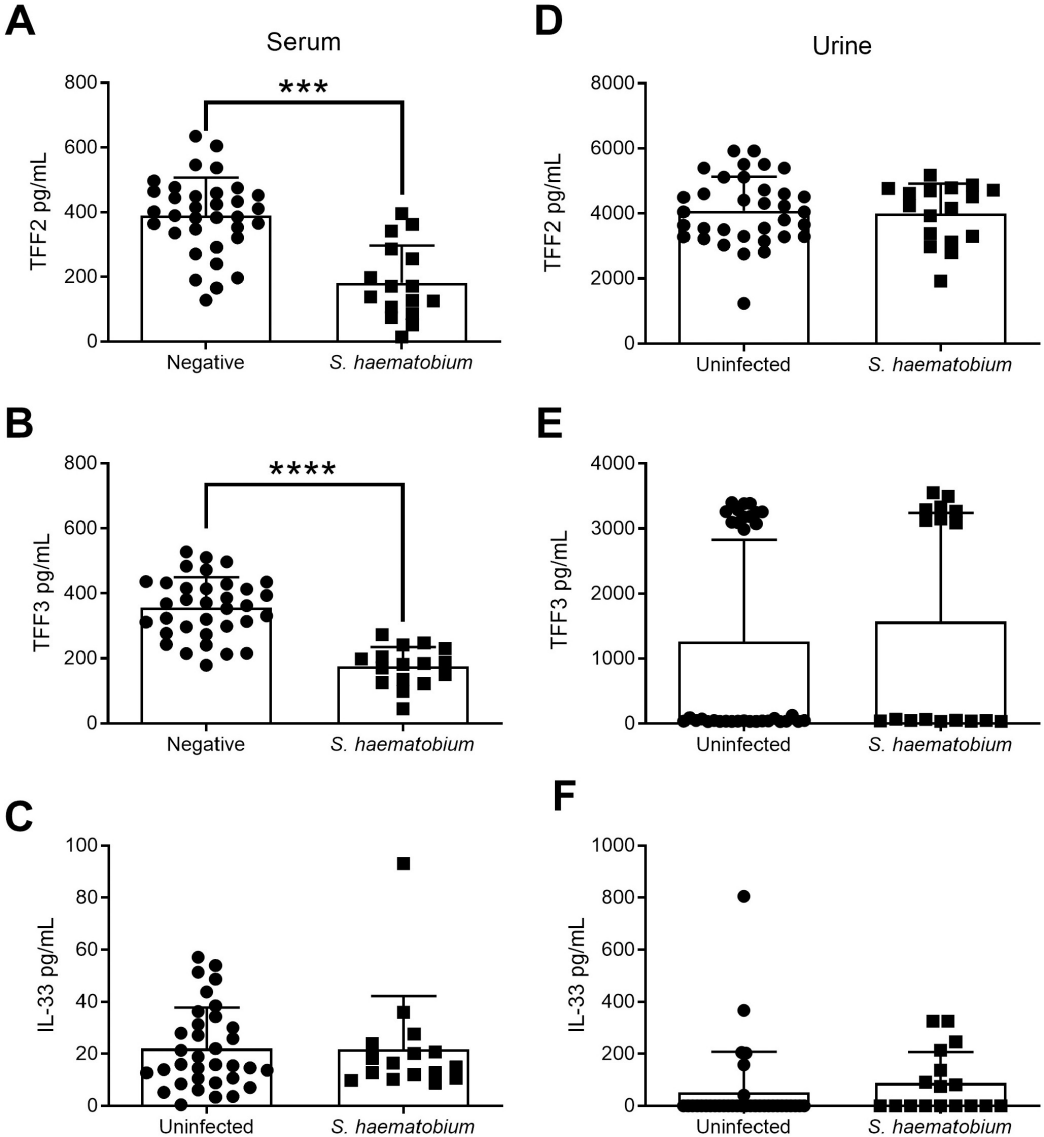
TFFs are decreased in serum but not urine samples from Nigerian children infected with *S*. *haematobium*. (A, B) Levels of TFF2 or (C, D) TFF3 in serum (A, C) and urine (B, D) samples from Nigerian school children infected with *S*. *haematobium* (n = 16–17) versus uninfected controls (n = 34). *** p<0.005, **** p<0.0001.

For TFF2, TFF3, and IL-33, we found both TFF2 (F test, p<0.0001) and TFF3 (F test, p = 0.0028) were significantly decreased in serum samples from children infected with *S*. *haematobium* (Fig 4A and 4B), but not IL-33 (Fig 4C). There was a strong association of age with serum levels of TFF2 and TFF3 (Table 5), and but the sample size within the infected and uninfected age groups restricted a formal analysis of age and infection interaction. TFF2 levels are much higher in urine than serum levels for the whole cohort, and TFF3 levels also seem to be higher in urine than serum in some cases. TFF2, TFF3, and IL-33 levels in urine were not significantly different between infected and uninfected children (Fig 4D–4F).

### *S*. *haematobium* infection is associated with more cytokine increases in urine than serum

**Fig 5:**
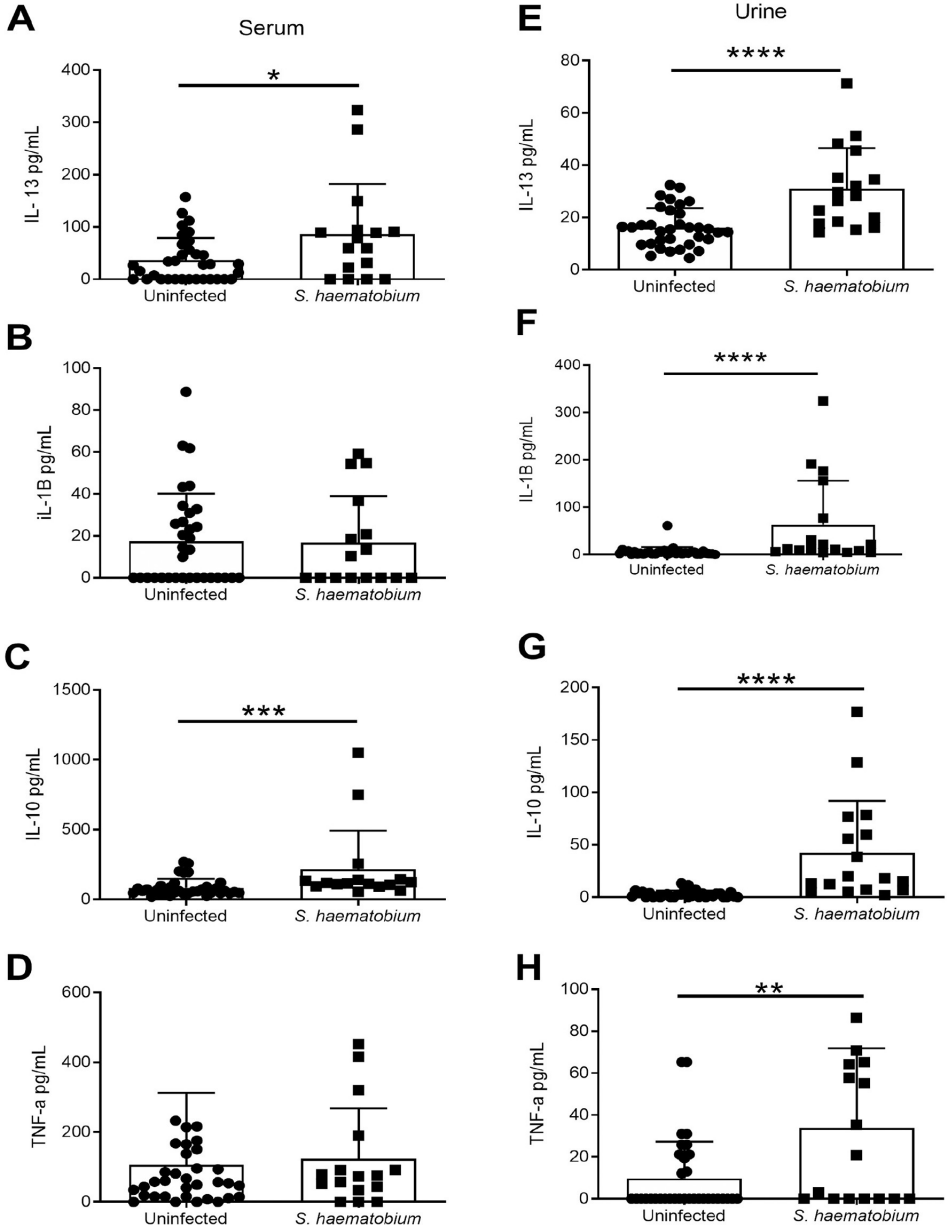
Cytokine (IL-13, IL-1b, IL-10, and TNF- α) levels in serum or urine samples of Nigerian children infected with or without *S*. *haematobium*. (A-D) Serum levels and (E-H) urine levels of (A, E) IL-13, (B, F) IL-1b, (C, G) IL-10, and TNF-α (D, H) were measured in infected (n = 16–17) and uninfected (n = 34) children. * p<0.05, ** p<0.01, *** p<0.001, **** p<0.0001.

**Fig 6:**
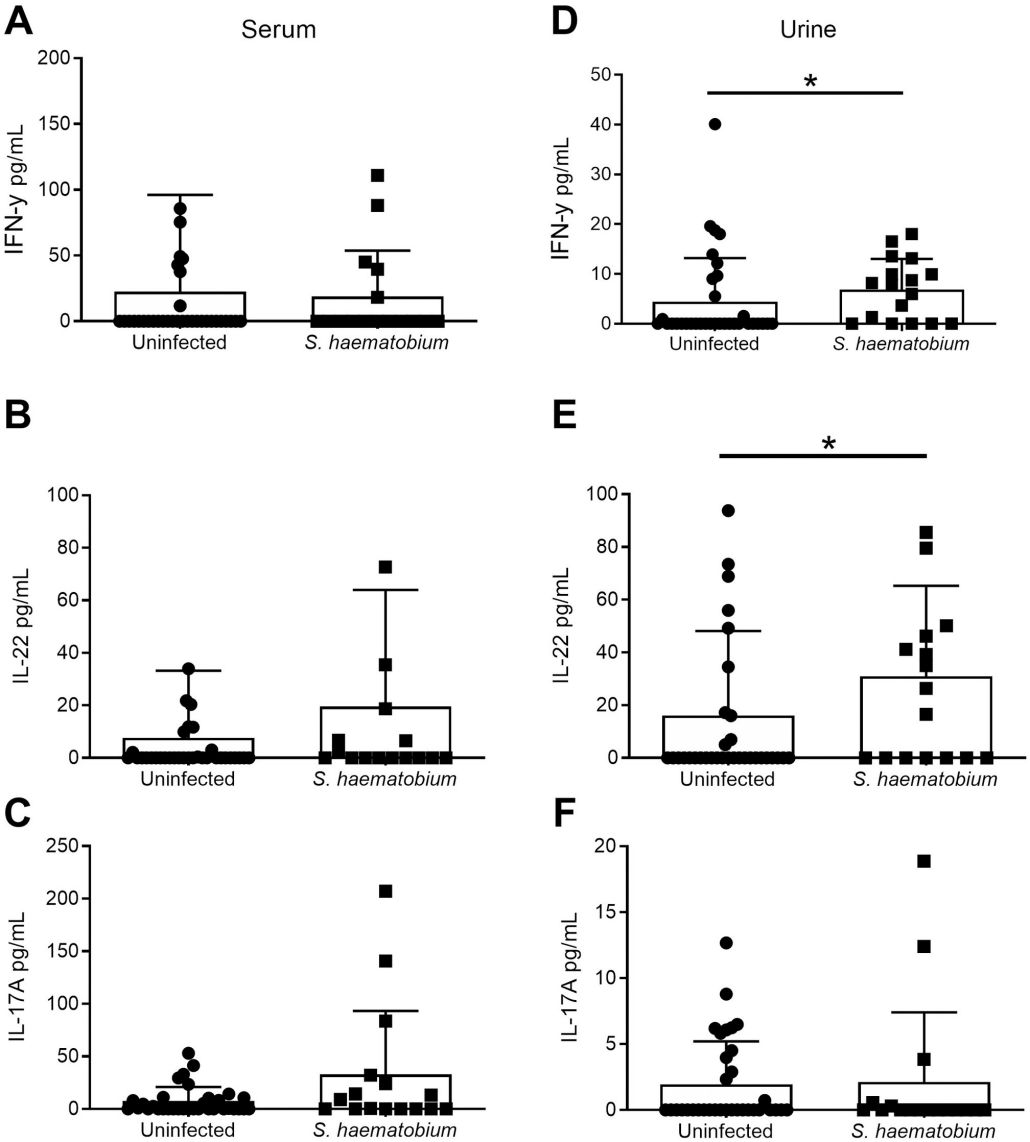
Cytokine (IFNγ, IL-22, and IL-17A) levels in serum or urine samples of Nigerian children infected with or without *S*. *haematobium*. (A-C) Serum and (D-F) Urine samples were measured for (A, D) IFN-γ, (B, E) IL-22, and (C, F) IL-17A levels in infected (n = 16–17) and uninfected (n = 34) children. * p<0.05.

We further evaluated consequences of *S*. *haematobium* infection on cytokine levels in serum and urine, where serum may reflect systemic inflammatory responses, while urine may reveal damage-associated signals as well as site-directed healing. Indeed, urine samples from children with *S*. *haematobium* infection had significantly elevated protein (0.07353 vs 1.029, t = 4.650, df = 16.74, p = 0.0002) and blood (0.1029 vs 1.765, t = 4.884, df = 17.54, p = 0.0001) as compared to uninfected controls, but across other general measurements of the urine samples (e.g. pH, specific gravity, etc.) did not differ significantly. Helminth infection is associated with alarmin cytokine production, and clearance is broadly associated with Type 2 immune responses [41]. In both compartments, we measured IL-1β, which is in the same family as IL-33 [42], but of distinct function and cytokines associated with proinflammatory immune responses and enhanced tissue damage (IFNγ, TNFα) [43], with Type 2 immune responses (IL-13) [41], and other regulatory or healing processes (IL-17A, IL-22, IL-10) [44,45]. As with TFF levels, serum and urine samples revealed differing patterns of increased cytokine levels in infected versus uninfected children. Serum levels were only significantly different for IL-10 (X^2^ = 11.6955, p = 0.0006) and IL-13 (non-zero data, X^2^ = 4.3105, p = 0.0379) (Fig 5). In urine, there was a significant increase in IL-13 (X^2^ = 15.9450, p<0.0001), IL-1β (X^2^ = 25.0070, p<0.0001), IL- 10 (X^2^ = 23.1514, p<0.0001), in infected versus uninfected children (Kruskal-Wallis Tests), and a significant association of infection with the presence of IL-22 (X^2^ = 4.0326, p = 0.0446), IFN-γ (X^2^ = 10.0388, p = 0.0015) and TNFα (X^2^ = 7.4009, p = 0.0065, Pearson Chi-Square Tests of Independence) (Figs 5 and 6). We found no difference between infected versus uninfected for levels of IL-17A in either sample type, or IL-22 in serum (Fig 6). Taken together, these data indicate that significant elevations in inflammatory cytokines (IL-13, IL-1β, TNF α, IL-22, IFN-γ) and the regulatory cytokine IL-10 are detected at the active site of damage in the urinary tract (urine), while only two (IL-13 and IL-10) are significantly changed in the serum.

## Discussion

Our previous work in mouse models of helminth infection strongly suggests that trefoil factor family proteins contribute to protective immune responses and parasite clearance [32–35]. To further support the translational potential of TFFs as a biomarker of infection or therapeutic target, we evaluated the levels of TFF2 and TFF3 for the first time in human helminth infection in geographically distinct regions. In Brazil, we found that human TFFs are modulated by helminth infection in a species, sex, and age-dependent manner. TFF2 was specifically elevated by hookworm infection in women and increased with age rather than parasite burden. TFF3 was significantly elevated by Schistosoma infection, and overall higher in women than men. In contrast, infection with the blood fluke *S*. *haematobium* decreased TFF2 and TFF3 levels in serum of infected Nigerian children, also with some evidence of age-dependence. Cytokines IL-10 and IL-1β were also significantly increased in the serum of infected children. There were increased levels of cytokines in the urine of infected children, including proinflammatory IL-1b, TNF-α, INF-γ, the type-2 cytokine IL-13, IL-22 and the inhibitory IL-10. Exogenous TFF2 was associated with suppressed TNF-α and IFN-γ production by stimulated human PMBCs, suggesting that elevated TFF2 in the context of hookworm infection could reduce these pro-inflammatory cytokine levels through direct or indirect mechanisms *in vivo*. Given that TFF2 is low in younger populations and further reduced in *S*. *haematobium* infected children, this could possibly explain why TNFα is high in urine and by proxy the infection site of the bladder and urinary tract, where *S*. *haematobium* eggs emerge from tissue into the urine. These findings bring us closer to understanding the mechanisms involved in human helminth infection. This information is necessary for uncovering diagnostic biomarkers to evaluate treatment efficacy and to develop better strategies to control infection.

While we were able to make advances by reporting infection, sex, and age-specific effects on TFF2 and TFF3 levels in human helminth infection, these studies do have some shortcomings that limit their interpretation. First, we were able to make an accurate assessment of hookworm egg counts in the Brazilian cohort, but a similar assessment for *S*. *mansoni* infected individuals was not as feasible, thus not included in this study to make a similar assessment for TFF3 and egg burden or age. Second, we were not able to collect enough sample in Brazil to also make cytokine assessment as performed in the Nigerian cohort, which was limited to children. This limits the ability to compare the patient cohorts. Lower sample size and different methodologies also limit the ability to draw firm conclusions regarding age and sex-related effects in the Nigerian data. Further, we made a preliminary attempt to determine what relationship human TFF might have on cytokine production of stimulated human PMBCs, but additional work is needed to demonstrate a direct effect of TFFs on cytokine levels in the context of human helminth infection, such as use of PMBCs from infected individuals or inclusion of helminth antigens in similar culture experiments. Despite these shortcomings, these findings provide first evidence of altered TFF levels in the context of human helminth infection in two different geographical regions.

It is generally accepted that tissue damage caused by the invading larval stages of various worm species drives prototypical Type 2 responses [46–50], which contribute to worm clearance and tissue repair [41]. These type 2 responses are initiated by the release of alarmin cytokines like IL-25, IL-33 or, thymic stromal lymphopoietin (TSLP) from damaged tissue [46–50]. We were unable to detect any significant differences in IL-33 levels in either human cohort, suggesting that this cytokine is not a good biomarker of infection. This finding does not necessarily negate a potential role for IL-33 in human immune responses to helminths, as it may be relevant at the infection site at specific times during the course of infection, which may not be adequately sampled by minimally-invasive methods.

Alarmin cytokines are key signals to activate tissue resident type 2 innate lymphocytes (ILC2s) and CD4+ TH2 cells, leading to their proliferation and release of both type 2 cytokines (IL-13 and IL-5) [49]. We were able to detect significant elevation in IL-13 associated with *S*. *haematobium* infection, particularly at the site of damage, supporting the idea that type 2 immune responses occur in the context of human infection. This may suggest that IL-13 in urine could serve as a non-invasive biomarker to track treatment efficacy. Additionally, *S*. *haematobium* is difficult to model in experimental animals, but some features of acute or sub-acute urogenital schistomiasis can be recapitulated in a mouse model system [51]. Our findings in human infection largely concur with earlier findings in the mouse model, specifically chronically elevated IL-13 and TNFα in the bladder tissue of egg-injected mice and only transient changes in IL-17 [51]. Some of the regulatory cytokine findings did differ between human data and the mouse model. Specifically, IL-10 elevation, but not TGFβ was associated with human infection, whereas the opposite was true in the mouse model [51], which may be due to a longer duration of infection in human subjects. This supports the translational relevance of the mouse model system, as well as a potential avenue to uncover mechanisms that could be targeted to boost immunity in humans.

These type 2 responses are thought to both augment clearance mechanisms against helminth colonization and also act to initiate tissue repair [41]. Host tissue repair would be advantageous for the parasite, as helminth survival depends on the host’s survival. TFF proteins are also critical for mucosal tissue repair [52], and may act upstream or in parallel with type 2 immune responses. Although our work in mouse models of hookworm infection suggests that both TFF2 and TFF3 contribute to worm clearance, tissue repair, and the suppression of proinflammatory cytokines [32–35], we found that TFF2 is specifically increased in the context of human hookworm infection and can reduce secretion of proinflammatory cytokine by human PMBCs. In the mouse hookworm infection model *N*. *brasiliensis*, TFF2 is required for induction of IL-33, early Type 2 cytokine responses, and contributes to clearance of worms from the gut [35]. We found that high TFF2 in human hookworm infection was not associated with higher IL-33 levels. This may suggest a species difference that could be worth examining further. The lack of IL-33 induction in humans may explain why the infection persists. Further exploration as to why TFF2 in mice can induce IL-33, but may not do so as strongly in humans could help identify mechanisms where human immunity could be boosted to improve worm clearance.

In contrast to hookworm infection, *S*. *hematobium* infection in children was associated with a decrease in both TFF2 and TFF3 in the serum, but not the urine. The reason why TFFs modulation is detectable in serum, but not urine, while cytokine differences were seen only in urine and not serum is not entirely clear. It could point to different responses of the host to the adult form found in vasculature versus the eggs encysted in the bladder wall and released into the urine [53]. This is the first time TFFs have been examined in the context of *S*. *hematobium* infection, but there is some link in the literature connecting TFFs to bladder [54–56]. Of note for our findings, cats with idiopathic cystitis, a painful condition characterized by hypercontractivity and frequent urination, have reduced levels of TFF2 in bladder biopsies compared to healthy controls [55]. It may be useful to determine if TFF levels correlate with measurements of bladder dysfunction or other aspect of *S*. *hematobium* infection in the future. Given the age and species specific-effects on TFF2 levels in serum of the Brazilian cohort, it will be necessary to expand these studies in children to additional age groups in the future.

## Conclusion

It is critically important that we find ways to identify helminth infected individuals using easily obtained samples and inexpensive techniques that can be implemented in endemic regions. One of these approaches is the identification of disease biomarkers. Our findings provide an initial step toward determining if TFFs or other immune mediators might serve as biomarkers for specific helminth infections. Disease biomarkers could be used to track the efficacy of drug treatments for parasite clearance. We could then focus expensive mass drug administration efforts on the most vulnerable populations, like children at risk for developmental delay, which will minimize risk of generating drug resistant parasites, particularly Schistosomes [15,57].

## Supporting information

S1 Fig IL-33 levels were not significantly affected by sex, age, or infection in serum samples of Brazilian subjects. (A, B) Levels of IL-33 and (C, D) transformation required for statistical analysis to compare cohorts (A,C sexes pooled, B,D sexes separated): Schistosoma (S, n = 11, 6 female), Hookworm (H, n = 20, 9 female), or both (S+H, n = 16, 8 female) versus uninfected controls (endemic, EC, n = 25, 13 female and non-endemic, NEC, n = 20, 10 female).
(TIF)

S2 Fig rhTFF2, but not rhTFF3 reduces PHA-evoked TNF alpha and INF gamma produced by human PMBCs in culture. (A) TNF alpha levels or (B) INF gamma levels produced by cultured PMBCs (4 donors provided cells plated across 9 wells at a density of 2.5 x 10^5^ cells/well) following treatment with media alone, media with rhTFF2 or rhTFF3 (25 ng/μL each, n = 4 wells, 1 well/donor), PHA (50μg) alone or PHA with rhTFF2 or rhTFF3 (n = 8 wells, 2 wells/donor). Pairwise comparisons as indicated: * p<0.05.
(TIF)
