# Supplementary figures and images for: Parasitic helminth infections in humans modulate Trefoil Factor levels in a manner dependent on the species of parasite and age of the host
Source: PLoS Negl Trop Dis. 2021 Oct 18;15(10):e0009550. doi: 10.1371/journal.pntd.0009550 (PMC8553090; doi:10.1371/journal.pntd.0009550)

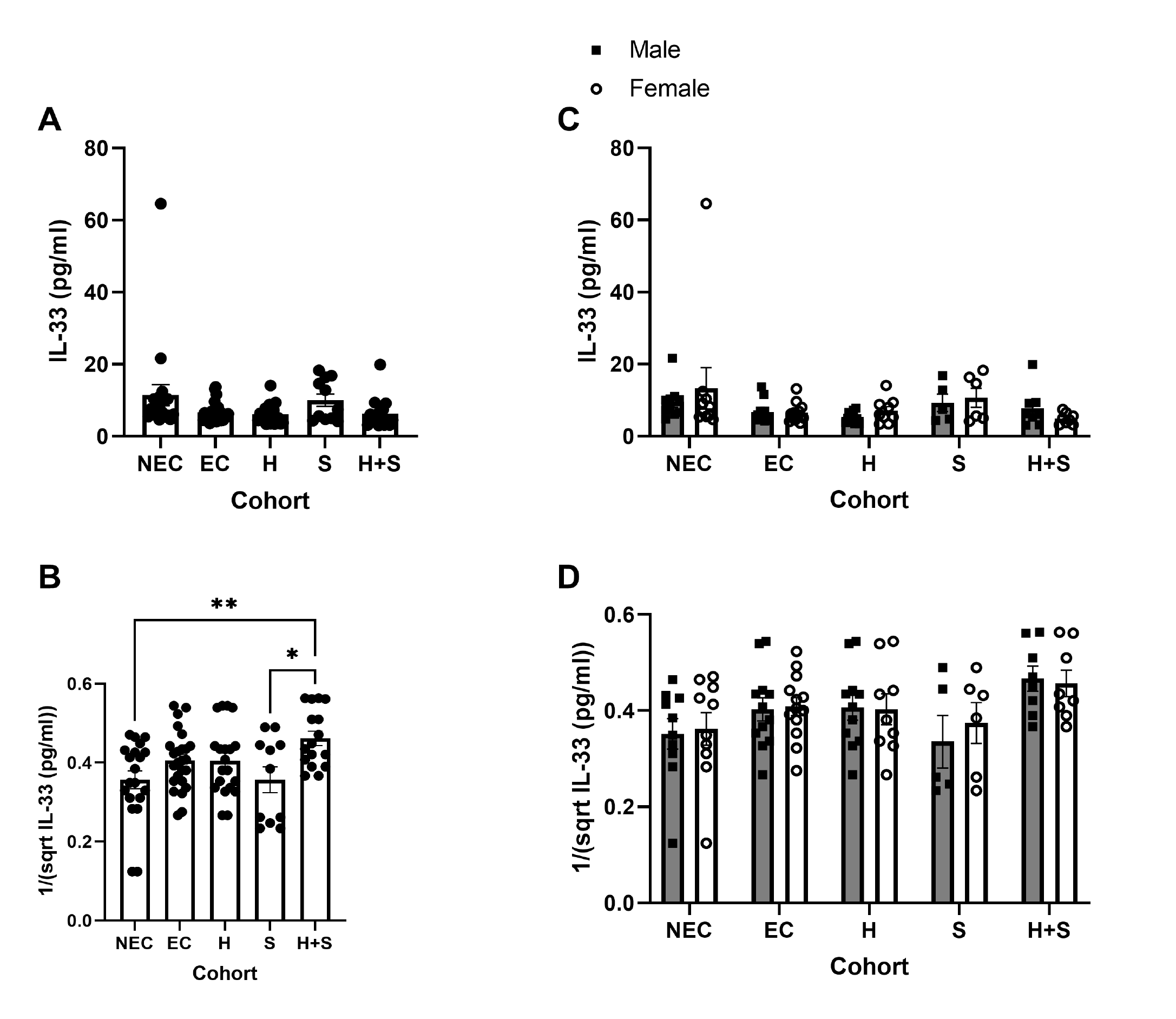

Supplement: S1 Fig — (A, B) Levels of IL-33 and (C, D) transformation required for statistical analysis to compare cohorts (A,C sexes pooled, B,D sexes separated): Schistosoma (S, n = 11, 6 female), Hookworm (H, n = 20, 9 female), or both (S+H, n = 16, 8 female) versus uninfected controls (endemic, EC, n = 25, 13 female and non-endemic, NEC, n = 20, 10 female). (TIF) [file pntd.0009550.s001.tif]

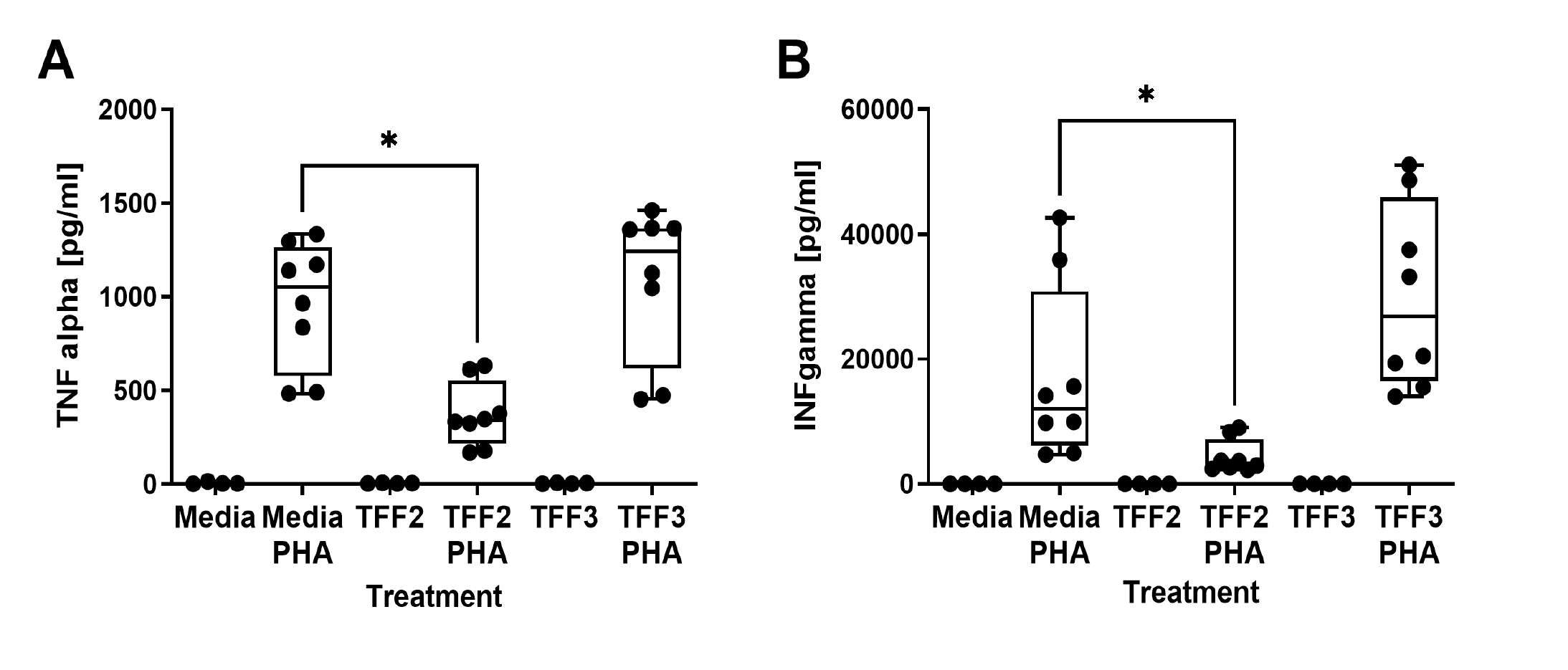

Supplement: S2 Fig — (A) TNF alpha levels or (B) INF gamma levels produced by cultured PMBCs (4 donors provided cells plated across 9 wells at a density of 2.5 x 10^5^ cells/well) following treatment with media alone, media with rhTFF2 or rhTFF3 (25 ng/μL each, n = 4 wells, 1 well/donor), PHA (50μg) alone or PHA with rhTFF2 or rhTFF3 (n = 8 wells, 2 wells/donor). Pairwise comparisons as indicated: * p<0.05. (TIF) [file pntd.0009550.s002.tif]
